# Towards advancement of nursing in Ghana: The role of the Ghanaian‐Diaspora Nursing Alliance (G‐DNA)

**DOI:** 10.1002/nop2.1997

**Published:** 2023-09-25

**Authors:** Thomas Hinneh, Foster Osei Baah, Evelyn Amoako, Diana Baptiste, Ruth‐Alma Turkson‐Ocran, Angela Agore, Stephen Adombire, Matilda Decker, Daniel Apau, Irene Fankah, Praba Koomson, Bernard Mensah, Jacqueline Idun, Samuel Akyirem, Yvonne Commodore‐Mensah

**Affiliations:** ^1^ Johns Hopkins University School of Nursing Baltimore Maryland USA; ^2^ Emory University School of Nursing Atlanta Georgia USA; ^3^ St. Dominic Hospital Akwatia Ghana; ^4^ Department of Medicine Research Section, Beth Israel Deaconess Medical Center/Harvard Medical School Boston Massachusetts USA; ^5^ King's College London UK; ^6^ Lawrence S. Bloomberg Faculty of Nursing University of Toronto Toronto Canada; ^7^ Lone Star College System Houston Texas USA; ^8^ Ghana College of Nurses and Midwives Accra Ghana; ^9^ Sutter Health Fairfield California USA; ^10^ University of Alberta Faculty of Nursing Edmonton Canada; ^11^ Yale School of Nursing, Yale University New Haven Connecticut USA; ^12^ Johns Hopkins Bloomberg School of Public Health Baltimore Maryland USA

Ghana, considered a lower‐middle income country (LMIC), has disparate levels of access to social services (e.g., health and education) compared with high‐income countries. These disparities shape the health outcomes, experiences, overall well‐being and quality of life of Ghanaians (Baah et al., 2019). The Ghanaian health system is plagued with several challenges including poor transportation systems, inadequate logistics and medical equipment and relevant human resource to enhance healthcare delivery (Sulemana & Dinye, 2014). Structural factors including the legacy of colonialism and urban biased health policy implementation also contribute to the differences in healthcare access in Ghana (Peprah et al., 2020). Furthermore, Ghana is experiencing a double burden of communicable and noncommunicable diseases, which threatens efforts to achieve the sustainable development goals. To equitably respond to the shortage of healthcare workers and address the burden of diseases, Ghana needs to train and support a strong nursing workforce with the capability to critique, evaluate and develop interventions to address the shifting healthcare needs of Ghanaians. Thus, we seek to describe the state of nursing education and practice in Ghana and highlight the role of *The Ghanaian‐Diaspora Nursing Alliance* (*G‐DNA*) in supporting efforts to enhance nursing education and practice to improve the health of Ghanaians.

## 1 STATE OF NURSING PRACTICE AND EDUCATION IN GHANA

In Ghana, nurses work throughout the healthcare pyramid providing care in varying settings. Nurses also serve as managers, quality improvement coordinators, counsellors and health educators (Afrose, 2017). The Ghana Government's flagship programme, Community‐based Health Planning and Services (CHPS) that seeks to enhance equity in health outcomes by decentralizing health services to remote and underresourced communities, is largely successful because nurses accept postings to rural communities without basic amenities (Government of Ghana, 2014). Indeed, about 80% of the healthcare needs of patients and populations are met by nurses, which illustrates the importance of nurses to the Ghanaian health system (Bassoumah et al., 2021).

Despite the significant contribution of nurses to healthcare delivery in Ghana, several challenges including limited professional development opportunities, poor working conditions, and inadequate remuneration hinder their ability to contribute fully to the delivery of healthcare in Ghana (Adu‐Gyamfi & Brenya, 2016). Between 2008 and 2018, the nursing and midwifery workforce increased by almost 370% due to a Human Resources for Health (HRH) strategy implemented by the Ministry of Health between 2007 and 2011 in response to the human resource crisis in the African region (Asamani et al., 2019). The 2021 Ghana Health Service Holistic Assessment Report showed that the number of nurses in Ghana particularly enrolled and community health increased from 44,167 in 2020 to 58,217 in 2021 (Asamani et al., 2019; Ghana Health Service, 2022). However, most of the nurses were trained for 2–3 years and were awarded diplomas mandating them to practice as general, community health and Enrolled Nurses (Nursing Assistants) (Asamani et al., 2019). Incontrovertibly, nursing care is safer, and evidence‐based, and leads to improved outcomes when delivered by nurses trained at the bachelor's degree level or higher (Harrison et al., 2019).

In the wake of the global nursing shortage, subsequently exacerbated by the COVID‐19 pandemic, countries are required to institute an effective and efficient nursing education program geared towards building a competent, well‐motivated and resourced nursing workforce. Despite the launch of the Plan of Action for Scaling up Quality Nursing and Midwifery Education and Practice for the African Region 2012–2022, progress made in nursing and midwifery education in Ghana and across Africa has stalled compared with the rising healthcare challenges on the continent (World Health Organization, 2021). Yet, the number of nursing and midwifery training institutions in Ghana has increased significantly in the last decade. Data available at the Nursing and Midwifery Council of Ghana (N&MC) as of June 2023 show that there are 135 nursing and midwifery training institutions accredited with the N&MC (Nursing and Midwifery Council of Ghana, 2023). Of those, 81 are state‐owned and the remaining 54 are privately owned institutions. Furthermore, 111 and 24 of the nursing and midwifery training institutions are diploma‐ and bachelor‐awarding institutions, respectively (Nursing and Midwifery Council of Ghana, 2023). While there are ongoing efforts to improve the quality of training and enhance nurses' professional competencies, there are still opportunities to establish global partnerships to strengthen nursing and midwifery education in Ghana.

## 2 GHANAIAN NURSES IN THE DIASPORA

Approximately 3000 professional nurses left Ghana in the first quarter of 2022 and between 400 and 500 nurses leave Ghana for developed countries almost every month (Ghana Registered Nurses and Midwives Association, 2023). Additionally, the number of Ghanaian‐trained nurses working in the UK increased by 1328 percent between 2019 and 2022, which is higher than nurses from any other African country (Royal College of Nursing, 2022). Ghanaian nurses (*N* = 746) were among the highest candidates for the 2022 NCLEX examination between January and December 2022. About 57.9% (432) of these candidates passed the examinations and are likely to have left Ghana for the United States (National Council of State Boards of Nursing, 2023). In a broader context, it is estimated that out of 2.3 million immigrant health workers in the United States alone, about 20% are registered nurses of which almost 24% were African‐born (Commodore‐Mensah et al., 2021). With the worsening economic crises in Africa and the accompanying effect of globalization, nurses' emigration from low‐resource settings may continue and seem unavoidable (Renzaho, 2016). The driving force behind the migration of nurses from Ghana needs to be interrogated thoroughly. As these Ghanaian nurses migrate to high‐income countries, there is a unique opportunity to establish collaborations with nurses in Ghana to advance nursing education and practice.

## 3 BACKGROUND OF THE GHANAIAN‐DIASPORA NURSING ALLIANCE (G‐DNA)

The G‐DNA is a non‐profit organization that seeks to harness the potential of Ghanaian nurses in the diaspora to enhance nursing education and practice in Ghana. Its mission is to ‘create local to global collaboration between nurses in Ghana and their counterparts in the diaspora’. In the spirit of collaboration, the G‐DNA has established formidable relationships with the Nursing and Midwifery Council (N&MC), the Ghana College of Nurses and Midwives (GCNM), the Ghana Registered Nurses and Midwives Association (GRNMA), the Ghana Health Service (GHS) and other public institutions offering nursing programmes in Ghana.

Workable solutions to improve nursing and midwifery education and practice have long been identified. ‘Curriculum reforms, profession regulation, transformative teaching strategies, collaboration and partnership, capacity building and infrastructure and resources’ are core thematic areas to improve the quality of Nursing and Midwifery Education and Practice in Ghana (World Health Organization, 2021). These areas of development are consistent with the World Health Organization Global Strategic Directions for Nursing and Midwifery (2021–2025) (World Health Organization, 2021). Ghanaian nurses who have acquired advanced training in high‐income countries have significant roles to play in nursing practice and education. The G‐DNA seeks to leverage the expertise of diaspora nurses across the globe to support nursing education, research and practice.

## 4 G‐DNA'S SPECIFIC AREAS OF ACTION

First, G‐DNA will collaborate with academic institutions in Ghana to advance curriculum development and support transformative teaching strategies. Ghanaian nurses in the diaspora are potential human capital to advance nursing education and practice in Ghana. To contribute to addressing the shortage of faculty members in nursing institutions, G‐DNA will connect qualified Ghanaian nurses in the diaspora to academic institutions to serve as faculty members based on their level of training and clinical experiences.

G‐DNA will support, empower and elevate Ghanaian diaspora nurses globally. Through these endeavours, G‐DNA aims at instituting and promoting continuing education programs, webinars and conferences to its members, and nurture a culture of lifelong learning. G‐DNA will continue to work with Ghanaian healthcare organizations, institutions and universities to expand their reach and impact on clinical nursing. These enduring efforts will fulfil G‐DNA's mission to foster sustainable local–global collaboration between Ghanaian nurses in Ghana and those in the diaspora to advance nursing education in Ghana and improve the health outcomes of Ghanaians. Mentorship and career guidance have been identified as a strategy to support nurses, especially early career professionals. G‐DNA believes that Ghanaian nurses in the diaspora can share knowledge and skills with their counterparts in Ghana, through intentional mentorship opportunities and virtual engagement activities such as educational webinars and conferences.

The G‐DNA plans to support the Ghana Health Service in organizing periodic local leadership forums and training for nurses in leadership positions. Nurses and midwives are seriously underrepresented at the institutional and national levels in decision‐making and policy formulation in Ghana. Though often overlooked, the perpetual lack of nursing presence during healthcare decision‐making and policy formulation has dire consequences on healthcare delivery. While the limited leadership training opportunities for nurses in Ghana may contribute to nurses' migration, the G‐DNA also believes that a major transformation in nursing and midwifery education with a focus on expanding the scope of nursing practice to include nurse leaders in policy formulation may improve service delivery and nurse retention.

## 5 CONCLUSION

The future of the nursing profession in Ghana and the entire Ghanaian healthcare system is inextricably linked to a strong, dedicated and well‐educated nursing workforce. This underscores an urgent need for a nursing workforce with diversity in training levels, clinical specialties, education and research skillsets to address the ever‐rising healthcare needs of the Ghanaian population, particularly the underserved and rural populations. We believe the G‐DNA is strategically positioned to work collaboratively with key stakeholders to not only meet this urgent need but more importantly, enhance healthcare delivery and access with an overall goal of improving the health of the Ghanaian population. To this end, G‐DNA looks forward to collaborating with the N&MC to reshape the future of the Ghanaian nursing profession.
